# Association between emotional intelligence and organizational commitment among clinical nurses: workplace social capital as a potential mediator in a cross-sectional study

**DOI:** 10.3389/fpsyg.2026.1871400

**Published:** 2026-10-13

**Authors:** Linfei Ding, Qiaohua Ye, Lihua Yu, Min Liao, Haixin Du, Yunxun Chen, Fengqiong Jiang

**Affiliations:** 1 Mianyang Central Hospital, School of Medicine, University of Electronic Science and Technology of China, Mianyang, China; 2 Dazhou Vocational College of Chinese Medicine, Dazhou, China

**Keywords:** emotional intelligence, mediating effect, nursing management, organizational commitment, workplace social capital

## Abstract

**Background:**

Workplace social capital has been shown to alleviate occupational stress and enhance job wellbeing and satisfaction. However, limited research has examined the mediational pattern involving workplace social capital between EI and organizational commitment among clinical nurses. This study aims to investigate the indirect effect of EI on organizational commitment through workplace social capital, providing empirical evidence for nursing managers to develop strategies that may enhance nurses' organizational commitment.

**Methods:**

A cross-sectional study design was adopted. A total of 648 clinical nurses from two tertiary and two secondary hospitals in Sichuan Province were recruited between June and November 2024 using a convenience cluster sampling approach. The research instruments included a general information questionnaire, the EI Scale, the Workplace Social Capital Scale, and the Organizational Commitment Scale. Descriptive statistics were performed using SPSS version 27.0, and structural equation modeling was conducted with AMOS version 24.0.

**Results:**

EI showed a significant direct positive association with organizational commitment after controlling for workplace social capital (effect = 0.229, *p* < 0.001; bias-corrected 95% CI [0.126, 0.342]), accounting for 38.62% of the total effect. In addition, the indirect association between EI and organizational commitment through workplace social capital was statistically significant (effect = 0.364, *p* < 0.001; bias-corrected 95% CI [0.295, 0.444]), accounting for 61.38% of the total effect. These findings are consistent with a partial mediation model.

**Conclusion:**

The findings of this cross-sectional study suggest that workplace social capital is an important intervening variable in the relationship between EI and organizational commitment among clinical nurses, with the indirect effect accounting for a larger proportion of the total effect than the direct effect. These results provide empirical support for the hypothesized mediating model, though causal conclusions require longitudinal or experimental validation.

## Introduction

Organizational commitment (OC), a core construct reflecting the psychological bond between employees and their organization, encompasses employees' identification with organizational values, the strength of their emotional attachment, and their intention to remain within the organization (Mowday et al., 1979). In nursing human resource management, a high level of OC not only serves as a key indicator of an organization's “magnetic” appeal but also substantially increases nurses' intention to stay and actual retention rates, making it a crucial predictor of turnover behavior among nurses (Lu and Zuo, 2021; Alshaibani et al., 2024; Labrague et al., 2018). Furthermore, multiple studies have demonstrated that OC is positively associated with a range of desirable outcomes among nurses, including innovative behavior, job performance, professional responsibility, and work engagement (Yu et al., 2013; Chu and Hsu, 2011). Consequently, exploring and promoting nurses' organizational commitment holds significant strategic value for stabilizing the nursing workforce and enhancing the overall quality of care.

Against this backdrop, emotional intelligence (EI)—defined as the ability to perceive, understand, and regulate one's own emotions as well as those of others—has increasingly been recognized as a critical psychological resource influencing nurses' organizational adaptation and the quality of their interpersonal interactions (Dulewicz and Higgs, 2000). Theoretical frameworks posit that EI is not a fixed trait but a set of multifaceted abilities that can be developed through learning and training (Law et al., 2004). Clinical nursing environments are characterized by high emotional labor and frequent interpersonal interactions (Bulmer Smith, 2009). According to EI theory, nurses with higher EI are better able to accurately recognize patients' emotional needs, regulate their own stress responses in high-pressure situations, and foster positive interactions with patients and colleagues through empathy and interpersonal management skills (Fteiha and Awwad, 2020; Mao et al., 2021). Such proficient emotional regulation may provide a fundamental foundation for nurses' successful professional adaptation and their ability to access effective social support within the organization.

However, nurses' psychological experiences within organizations are shaped not only by their individual capabilities but also by the social networks in which they are embedded (Tagoe and Quarshie, 2017; Pesut, 2002). Workplace social capital (WSC) represents a central manifestation of these networks; it refers to the interpersonal structures built upon shared norms, trust, and reciprocity, constituting a valuable resource embedded within the organizational fabric (Kouvonen et al., 2006). Work environments with high social capital are typically characterized by strong mutual trust, close social ties, and voluntary cooperative behaviors among members (Pesut, 2002). This raises a critical question: through which social mechanisms does EI ultimately influence OC? Social exchange theory offers a valuable framework for understanding this process, positing that much of human social behavior can be conceptualized as an implicit, reciprocity-based exchange system. When individuals receive socioemotional resources from their organization or colleagues—such as trust, respect, or support—they are likely to develop a sense of obligation to reciprocate (Cropanzano and Mitchell, 2005). Specifically, nurses with high EI, leveraging their acute empathy and exceptional interpersonal skills, are more adept at proactively building and maintaining positive social networks in the workplace (Akerjordet and Severinsson, 2007). Through the continuous provision of emotional support and collaborative behaviors, they may be associated with higher WSC and may reinforce reciprocal exchange, which may help explain stronger belonging and commitment (Hsu et al., 2011). Therefore, WSC may serve as a potential indirect pathway linking EI and OC, helping explain the association by transforming individual emotional competence into a stable psychological connection with the organization.

Based on the principle of reciprocity in social exchange theory (Cropanzano and Mitchell, 2005), WSC may be positively associated with OC; when nurses are situated within highly trusting and mutually supportive work networks and consistently receive emotional resources and practical assistance from them, such experiences are associated with stronger affective identification and attachment to the organization. Empirical research also supports this perspective: for instance, a cross-sectional study conducted among nurses in Iran found that both the cognitive and structural dimensions of WSC were significantly positively correlated with affective commitment, suggesting that greater connections and mutual support among nurses were associated with the promotion of shared goal-setting and affective commitment (Vagharseyyedin et al., 2018). Furthermore, research on the association between authentic leadership and nurses' organizational commitment suggests that workplace social capital may help explain this association, a pattern consistent with a potential role of social capital in nurses' organizational commitment (Kida et al., 2023).

In summary, the present study integrates EI theory and social exchange theory to develop a mediation model (Dulewicz and Higgs, 2000; Cropanzano and Mitchell, 2005). We hypothesize that clinical nurses' EI is positively associated with their OC, and that this relationship is partly indirect, operating through the accumulation of WSC. This study aims to systematically examine the relationships among EI, WSC, and OC in clinical nurses, with particular emphasis on the indirect pathway through WSC. Through this investigation, we seek to examine the mediational pattern linking emotional factors and OC via social factors, thereby providing empirical evidence that may inform human resource management strategies in nursing. Based on this framework, the study proposes the following hypotheses:

H1: Clinical nurses' EI is positively associated with OC.
H2: Clinical nurses' EI is positively associated with WSC.
H3: WSC is positively associated with OC.
H4: The indirect association of EI with OC through WSC is statistically significant, consistent with a partial mediation model.

## Method

### Study design

This study employed a cross-sectional survey design. From June to November 2024, a survey was conducted in two tertiary hospitals and two secondary hospitals in Sichuan Province. In-service nurses were recruited using convenience cluster sampling and surveyed by means of a questionnaire. The selection of participating hospitals took into consideration different hospital grades and was based on the research team's existing professional collaborations with the hospitals.

The sample size was determined according to the estimation method proposed by Kendall (Chen and Bu, 2000), which recommends that the sample size should be at least 5–10 times the number of variables included in the analysis. This study included three scales assessing EI, workplace social capital, and organizational commitment, comprising a total of 42 items. Based on the 10:1 criterion, the minimum required sample size was calculated to be 420 participants.

### Participants

The sampling procedure was implemented in two stages. First, nursing units directly involved in providing clinical care in each hospital were identified as the primary sampling clusters. Second, after obtaining approval from hospital administrative departments, the researchers distributed questionnaires to nurses during their regularly scheduled unit meetings, which served as the venue for on-site data collection. The inclusion criteria were as follows: (1) registered nurses holding a valid nursing license and currently employed in clinical practice; (2) a minimum of 1 year of clinical nursing experience; and (3) the ability to fully understand the study and a willingness to participate voluntarily. The exclusion criteria were as follows: (1) nurses on maternity or sick leave at the time of recruitment; (2) nurses working in non-clinical units, including auxiliary departments, logistics, supply rooms, or health management centers; and (3) nurses engaged in further education, residency training, or clinical rotations.

## Measures

### General information questionnaire

A self-developed general information questionnaire was used in this study, encompassing 11 sociodemographic and occupational variables: gender, age, hospital level, department, average monthly income, educational background, job position, marital status, employment type, night shift status, and type of household registration.

### EI scale

The EI Scale, developed by Wong and Law (2002) (Wong and Law, 2002), comprises 16 items across four dimensions: Self-Emotion Appraisal (SEA), Others' Emotion Appraisal (OEA), Use of Emotion (UOE), and Regulation of Emotion (ROE). Wang (2010) translated and validated the Chinese version of the scale. The Chinese version maintains the identical 16-item structure and four-dimensional framework as the original English version. In the validation study, the overall Cronbach's α was 0.915, with subscale α values ranging from 0.692 to 0.802. Items are rated on a 7-point Likert scale (1 = “strongly disagree” to 7 = “strongly agree”), yielding total scores from 16 to 112. Higher scores indicate greater EI. In the present study, the overall Cronbach's α was 0.903, with subscale α values ranging from 0.831 to 0.881, indicating high internal consistency.

### Workplace social capital scale

The Workplace Social Capital Scale was originally developed by Kouvonen et al. in 2006 and later translated and culturally adapted to the Chinese context by Zhang et al. (2014). The revised version comprises 8 items divided into two dimensions: linking Social Capital (3 items, referring to vertical interactions with supervisors) and Non-Linking Social Capital (5 items, referring to horizontal interactions with colleagues). Items are rated on a 5-point Likert scale (1 = “strongly disagree” to 5 = “strongly agree”), yielding total scores from 8 to 40. In the original study, the overall Cronbach's α was 0.898, with subscale α values of 0.845 and 0.839. In the present study, the overall Cronbach's α was 0.941, with subscale α values of 0.882 and 0.919, indicating good internal consistency.

### Organizational commitment scale

The Organizational Commitment Scale, developed by Allen and Meyer (1990), comprises 18 items across three dimensions: Affective Organizational Commitment (AOC), Continuance Organizational Commitment (COC), and Normative Organizational Commitment (NOC). These three dimensions capture the desire (AOC), need (COC), and perceived obligation (NOC) to stay with the organization, respectively. In 2000, Lin Yuanji translated and culturally adapted the scale to develop the Chinese version (Gui et al., 2013). Items are rated on a 5-point Likert scale (1 = “disagree” to 5 = “strongly agree”), yielding total scores from 18 to 90. Higher scores indicate greater OC. In the present study, the overall Cronbach's α was 0.885, with subscale α values ranging from 0.770 to 0.880, indicating good internal consistency.

## Ethical approval

Ethical approval for this study was granted by the Ethics Committee of Mianyang Central Hospital before data collection (Approval No.: S20240383-01).

## Data collection

A mixed online–offline questionnaire survey was employed. In the tertiary hospital located in Mianyang, paper questionnaires were distributed and collected in person. The research team contacted the head nurses of each department in advance and administered the questionnaires during monthly nursing meetings. For hospitals outside Mianyang, questionnaires were distributed online via the Questionnaire Star (Wenjuanxing) platform, following approval from each hospital's nursing department and with assistance from the head nurses. A total of 800 questionnaires were distributed. After quality control, 152 invalid questionnaires were excluded (136 with patterned responses and 16 with missing key data), yielding 648 valid responses. Of these, 300 and 197 were from the two tertiary hospitals, and 81 and 70 from the two secondary hospitals. Based on estimated nursing staff totals at each site (approximately 900, 800, 400, and 400, respectively), the hospital-level valid responses (valid responses / total nurses) were approximately 33%, 25%, 20%, and 18%.

## Data analysis

This study used SPSS 27.0 for descriptive statistics, reliability analysis, and Pearson correlations. As a supplementary analysis, hierarchical multiple linear regression using ordinary least squares (OLS) was conducted to assess the robustness of the associations of EI and WSC with OC after accounting for demographic and occupational characteristics, including hospital level. Model 1 included EI and WSC, and Model 2 additionally included these covariates. Regression coefficients, ΔR^2^, and statistical significance were examined. Multicollinearity, residual independence, linearity, homoscedasticity, residual distribution, and potential outliers were assessed using standard diagnostic procedures.

Given the clustered sampling, we conducted a leave-one-hospital-out sensitivity analysis by sequentially excluding each hospital to assess robustness. Common method bias was tested using Harman's single-factor test and the unmeasured latent method factor approach.

Structural equation modeling (SEM) was performed using AMOS 24.0. Since skewness (<3) and kurtosis (<10) indicated acceptable univariate normality, maximum likelihood (ML) estimation was used. Confirmatory factor analysis (CFA) evaluated construct validity via composite reliability (CR) and average variance extracted (AVE), and discriminant validity by comparing the square root of the AVE with inter-construct correlations. Multi-group CFA tested configural and metric invariance across hospitals (ΔCFI ≤ 0.01). Model fit was assessed using χ^2^/df, RMSEA, SRMR, CFI, TLI, NFI, IFI, and GFI, with acceptable thresholds of χ^2^/df <5, RMSEA/SRMR <0.08, and the other indices > 0.90. Bootstrapping (2000 resamples) provided bias-corrected 95% CIs for path coefficients. All tests were two-sided, with *p* < 0.05 considered significant.

## Results

### Common method bias

Several procedural remedies were implemented during the data collection phase to mitigate the risk of common method bias (CMB). Specifically, respondent anonymity was ensured, participants were explicitly informed that there were no right or wrong answers, and the independent and dependent variables were separated into distinct sections of the questionnaire. Collectively, these measures helped reduce the potential for CMB at its source. Harman's single-factor test was employed to evaluate potential CMB. Nine factors with eigenvalues greater than 1 were extracted without rotation, with the first factor accounting for 30% of the total variance—below the critical threshold of 40%. The results of the unmeasured latent method factor technique indicate that the path coefficients among the core variables in the model varied within a range of 0 to 0.03 (<0.05) (Appendix 1) (McDonald and Ho, 2002). Accordingly, CMB was deemed unlikely to have substantially influenced the study results.

### General information

The detailed sociodemographic and occupational characteristics of the clinical nurses are summarized in Table 1.

**Table 1:** General demographic and occupational characteristics of participants.

Category	*n*(%)	Category	*n*(%)
Gender		Highest Educational Attainment	
Male	30(4.6)	Diploma or below	99(15.3)
Female	618(95.4)	Bachelor's degree or above	549(84.7)
Age (years)		Position	
<25	68(10.5)	None	517(79.8)
26–35	400(61.7)	Team leader	84(13)
36–45	144(22.2)	Head nurse or above	47(7.3)
>46	36(5.6)		
Hospital grade		Marital Status	
Secondary hospital	151(23.3)	Unmarried	145(22.4)
Tertiary hospital	497(76.7)	Married	503(77.6)
Department		Employment Type	
Internal medicine	244(37.7)	Contract-based	478(73.8)
Surgery	174(26.9)	Public institution staff	170(26.2)
Obstetrics and Gynecology	42(6.5)	Night Shift Frequency	
Pediatrics	57(8.8)	No night shifts	129(19.9)
Emergency	35(5.4)	<2times/month	84(13)
Outpatient	37(5.7)	3–6times/month	166(25.6)
ICU	59(9.1)	>7times/month	269(41.5)
Monthly income (CNY)		Household Registration	
<5000	135(20.8)	Local (city or county)	424(65.4)
5001–8000	255(39.4)	Within the province	207(31.9)
8001–10000	211(32.6)	Outside the province	17(2.6)
>10001	47(7.3)		

### EI, Workplace social capital, and organizational commitment among clinical nurses

The total EI score of clinical nurses was 78.13 ± 12.77, with a mean item score of 4.88 ± 0.80. Across the four dimensions, mean item scores ranked from highest to lowest as SEA, OEA, UOE, and ROE. The total WSC score was 31.79 ± 5.51, with a mean item score of 3.97 ± 0.69. Scores for linking social capital were slightly higher than those for non-linking social capital. The total OC score was 64.20 ± 10.40, with a mean item score of 3.57 ± 0.58. Among the three dimensions, scores ranked from highest to lowest as AOC, NOC, and COC. The absolute skewness values for all three scales and their subdimensions were <3, and absolute kurtosis values were <10, indicating approximate normal distribution of the data (McDonald and Ho, 2002). Detailed results are presented in Table 2.

**Table 2:** Descriptive statistics and normality tests of each variable.

Construct	Number of items	Mean ±SD	Mean per item	Skewness	Kurtosis
**EI**	16	78.13 ±12.77	4.88 ± 0.80	−0.044	−0.301
SEA	4	22.51 ± 3.65	5.63 ± 0.91	−0.996	3.119
OEA	4	19.10 ± 4.18	4.77 ± 1.05	−0.170	0.088
UOE	4	18.48 ± 4.27	4.62 ± 1.07	−0.219	0.037
ROE	4	18.05 ± 4.74	4.51 ± 1.19	−0.134	−0.165
**WSC**	8	31.79 ± 5.51	3.97 ± 0.69	−0.706	0.623
LSC	3	11.97 ± 2.48	3.99 ± 0.83	−0.828	0.755
NON-LSC	5	19.81 ± 3.52	3.96 ± 0.70	−0.753	0.874
**OC**	18	64.20 ± 10.40	3.57 ± 0.58	−0.308	0.327
AOC	6	23.42 ± 4.43	3.90 ± 0.74	−0.600	0.364
NOC	6	20.81 ± 4.84	3.47 ± 0.81	−0.235	−0.006
COC	6	19.97 ± 4.08	3.33 ± 0.68	−0.299	0.573

### Correlation analysis among clinical nurses' EI, workplace social capital, and organizational commitment

Pearson's correlation analysis revealed significant positive associations among the three variables. Detailed results are presented in Table 3.

**Table 3:** Bivariate correlations between variables.

Variable	EI	WSC	OC
EI	1		
WSC	0.48^**^[0.42,0.53]	1	
OC	0.47^**^[0.41,0.53]	0.58^**^[0.52,0.62]	1

^**^P < 0.01.

### Measurement invariance testing

The multi-group confirmatory factor analysis showed that both the configural invariance model (χ^2^ (48) = 141.392, CFI = 0.955, RMSEA = 0.055) and the metric invariance model (χ^2^ (54) = 149.559, CFI = 0.954, RMSEA = 0.052) demonstrated acceptable fit. The change in CFI was 0.001, and the chi-square difference test was not significant (Δχ^2^ (6) = 8.168, *p* = 0.226), suggesting that metric invariance across hospital levels was supported (see Appendix 2 Table 8).

### Structural equation model construction

As a supplementary robustness analysis, hierarchical multiple linear regression analyses were performed. Model 1 included EI and WSC, while Model 2 additionally adjusted for 11 demographic and occupational characteristics, including hospital level. After adjustment, EI (*B* = 0.167, *P* < 0.001) and WSC (*B* = 0.828, *P* < 0.001) remained significantly associated with OC, with the directions unchanged. Detailed results are presented in Appendix 2 Table 1.

Sensitivity analyses showed that the associations of EI and WSC with OC remained statistically significant after sequentially excluding the sample from each hospital, suggesting that the core findings were generally robust and were not substantially affected by hospital level or the sample from any single hospital (see Appendix 2 Table 9).

Measurement Model Assessment (see Appendix 2: Model Fit Results for the Measurement Models of the Three Scales): Before constructing the structural equation model, confirmatory factor analysis was first conducted to assess the measurement models of each scale. The results showed that the overall model fit was acceptable for the EI Scale (χ^2^/df = 4.620, CFI = 0.942, RMSEA = 0.075) and the Workplace Social Capital Scale (χ^2^/df = 5.516, CFI = 0.986, RMSEA = 0.080). The Organizational Commitment Scale demonstrated an overall good model fit (χ^2^/df = 3.804, CFI = 0.940, RMSEA = 0.066); however, the discriminant validity between the normative commitment and affective commitment dimensions did not meet the ideal criterion based on the Fornell–Larcker criterion. Given the theoretically high correlation between these two dimensions and the acceptable overall fit of the measurement model for OC, OC was subsequently modeled as an overall higher-order construct in the structural model, consistent with its theoretically multidimensional construct structure. Overall, the convergent validity of the scales was supported. The composite reliability (CR) values for all latent variables were greater than 0.70, and the average variance extracted (AVE) values for all dimensions exceeded 0.50, except for the continuance commitment dimension of OC.

A structural equation model was constructed using AMOS version 24.0, with e EI specified as the exogenous latent variable, WSC as the mediating variable, and OC as the endogenous latent variable. The standardized path coefficients of the model are presented in Figure 1, and the model fit indices are presented in Table 4. All fit indices met the recommended criteria, indicating that the model demonstrated a good overall fit. The local model fit results are presented in Appendix 2 Tables 10, 11.

**Figure 1:**
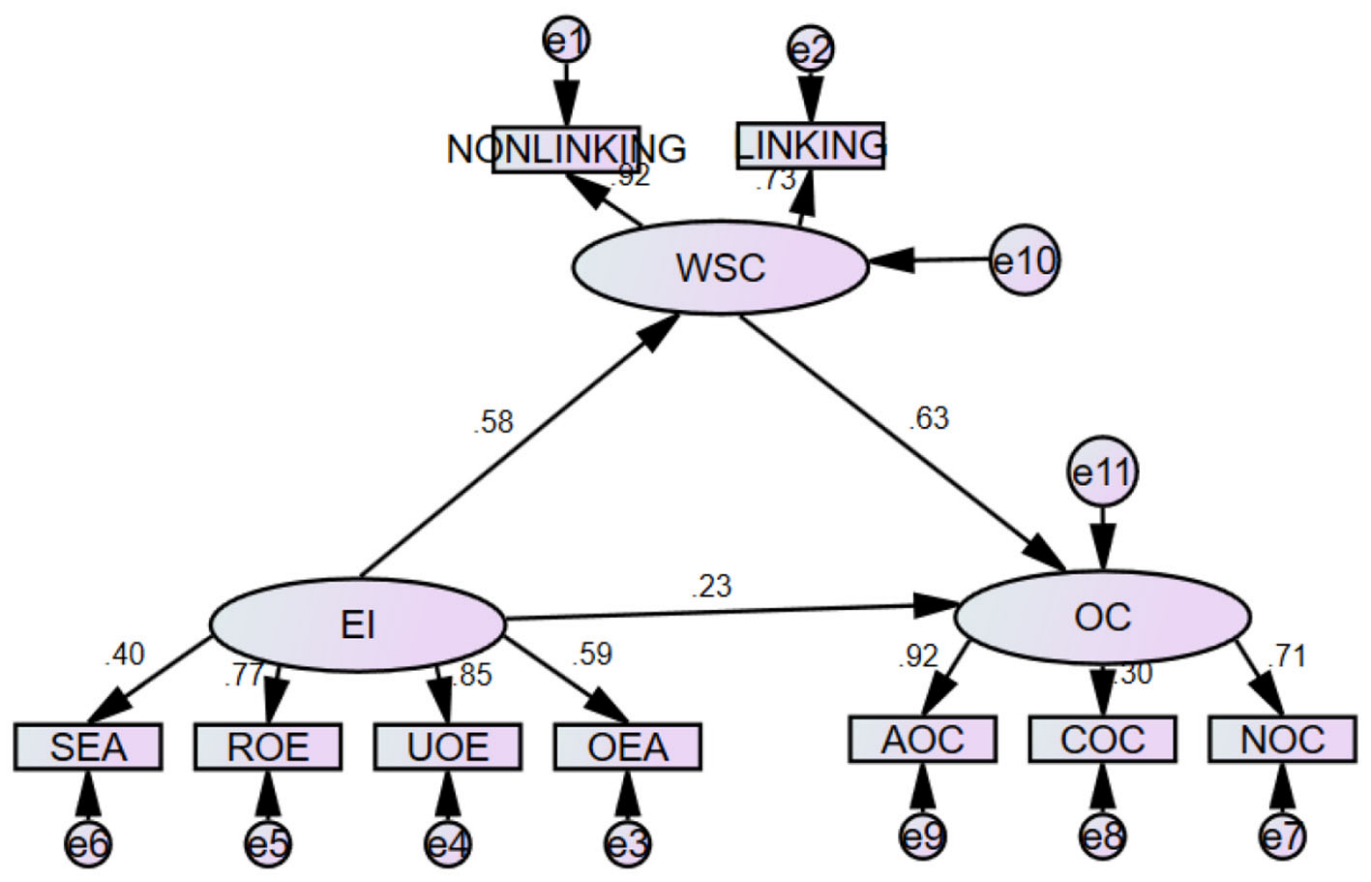
Structural equation model of EI, workplace social capital, and organizational commitment.

**Table 4:** Structural equation model fit results.

Fit index	Reference value	Measured value	Conclusion
χ^2^ (Chi-square)	—	84.368^**^	—
df (Degrees of Freedom)	—	24^**^	—
χ^2^/df	<5.00	3.515	Good fit
*P*-value	>0.05	<0.001	—
RMSEA	<0.08	0.062	Good fit
RMSEA 90% CI	—	[0.048, 0.077]	—
SRMR	<0.08	0.038	Good fit
CFI	>0.90	0.972	Good fit
TLI	>0.90	0.957	Good fit
NFI	>0.90	0.961	Good fit
IFI	>0.90	0.972	Good fit
GFI	>0.90	0.972	Good fit

^**^Indicates the default model in AMOS output, not statistical significance.

### Results of the mediation analysis

The non-parametric bootstrap method was applied to test the mediation effect. Bias-corrected 95% confidence intervals (CIs) for all three effect types excluded zero, indicating that WSC partially mediated the relationship between EI and OC. The mediating effect accounted for 61.38% [0.475, 0.776] of the total effect (0.364/0.593). Detailed results of the mediation analysis are presented in Table 5.

**Table 5:** Mediation analysis of workplace social capital between EI and organizational commitment.

Path	Effect type	Effect value	Bias-corrected 95%CI			Proportion of total effect	Conclusion
			Lower	Upper	*p*		
EI → WSC → OC	Total Effect	0.593	0.509	0.674	<0.001	100%	Partial Mediation
	Direct Effect	0.229	0.126	0.342	<0.001	38.62%	
	Indirect Effect	0.364	0.295	0.444	<0.001	61.38%	

## Discussion

### The current status of EI, workplace social capital, and organizational commitment among clinical nurses

The results indicated that the total EI score among clinical nurses was 78.13 ± 12.77, which is above the scale's theoretical midpoint of 64. Since this value falls within the upper half of the possible scoring range, we descriptively define this as a moderately high level. This finding aligns with prior studies by Song (2013), Yang (2018), Wang (2022), Xiao et al. (2021), Zhao et al. (2023), Zhang et al. (2010), and Abualruz et al. (2024). A plausible explanation is that clinical nursing entails emotionally demanding work, which may enhance nurses' ability to recognize and regulate emotions through sustained practice, thereby improving their overall EI. Among the four dimensions of EI, SEA had the highest score (22.51 ± 3.65), consistent with the findings of Yang (2018), Xiao et al. (2021), Zhao et al. (2023), and Abualruz et al. (2024). This may suggest that clinical nurses possess strong self-awareness of emotions, enabling them to avoid overt expressions of emotional conflict. The remaining dimensions, in descending order, were OEA, UOE, and ROE. The relatively high OEA score may stem from the profession's high demands for responsibility and empathy, as frequent interactions with patients and their families strengthen nurses' capacity to recognize others' emotions (Mohammad et al., 2021). By contrast, ROE had the lowest score, indicating challenges in emotional regulation. This may be linked to the complex interpersonal dynamics and multiple stressors encountered at work, involving patients, families, colleagues, and supervisors (McQueen, 2004). The score for the UOE was slightly higher than that for the ROE. Previous studies have shown that EI is positively associated with nurses' psychological resilience (Safi-Keykaleh et al., 2026). A higher ability to use emotions may facilitate the transformation of emotional resources into motivation that promotes personal growth and work performance (Abualruz et al., 2024).

Regarding WSC, the total score was 31.79 ± 5.51, exceeding the scale midpoint of 24, which similarly corresponds to a moderately high level according to the same criterion. Specifically, the score for linking social capital was 11.97 ± 2.48 (mean item score: 3.99 ± 0.83), while non-linking social capital scored 19.81 ± 3.52 (mean item score: 3.96 ± 0.70). These results are consistent with studies by Wu et al. (2021), Yang et al. (2022), Hou (2017), Xu et al. (2021), Berthelsen et al. (2021), Kida et al. (2024), and Fujita et al. (2016). Empirically, this relatively high level may be partly attributable to the collaborative structure of clinical nursing practice, which necessitates frequent interaction and mutual support among team members (Li et al., 2023). In addition, nurse managers' leadership styles are closely associated with WSC (Kida et al., 2023). Transformational leadership, through encouragement and support, fosters an open and trusting work environment, which is conducive to the development of social capital.

In terms of OC, the total score was 64.20 ± 10.40, surpassing the midpoint of 54 and thus also reflecting a moderately high level based on this theoretical benchmark. Scores for the three dimensions were: AOC = 23.42 ± 4.43, NOC = 20.81 ± 4.84, and COC = 19.97 ± 4.08. These findings are consistent with studies by Du (2022), Qu (2022), Yang (2021), Cui (2018), and El Said Ahmad et al. (2020). AOC had the highest score, which may be related to the fact that the majority of nurses in this study were from tertiary hospitals. Tertiary hospitals generally have more well established systems for career development, continuing education, and organizational support (Wang et al., 2025), which may provide nurses with greater opportunities for professional growth and organizational support, thereby strengthening their emotional attachment to and identification with the organization. NOC ranked second, suggesting a strong sense of professional responsibility and adherence to occupational norms, reflecting stability and professionalism (Li, 2010). COC was lowest. possibly because of the high work pressure and elevated occupational mobility of frontline nurses (Huyghebaert et al., 2019). In a favorable job market, ample career opportunities may reduce long-term commitment to their current institutions (Xu and Fang, 2007).

### The relationship between EI and workplace social capital

The findings of this study reveal a significant positive correlation between clinical nurses' EI and WSC (*P* < 0.01), a result that can be interpreted within the framework of EI theory. According to this theory, EI comprises four dimensions: perceiving, understanding, utilizing, and managing emotions (Mayer et al., 2003). Nurses with higher EI can accurately recognize both their own and others' emotions and subsequently engage in effective emotional expression and regulation, which may be associated with more harmonious interpersonal relationships in complex health-care settings (Xiao et al., 2021; Schutte et al., 2001). These capabilities may be associated not only with stronger emotional resonance and perceived trust within the team but also with greater participation in team collaboration and supportive interactions, patterns consistent with a positive social network grounded in reciprocity and trust (Zhang and Huang, 2015). Watkins et al. emphasized that high-quality interpersonal interactions constitute a critical foundation for the accumulation of WSC (Watkins et al., 2022). Furthermore, nurses with high EI tend to exhibit stronger emotional resilience and coping abilities when dealing with work-related stress or adverse events, which may be associated with a weaker negative association between emotional exhaustion and collegial relationships (Fteiha and Awwad, 2020; Zhao et al., 2023). Consequently, they may be more likely to access social resources both horizontally (among peers) and vertically (between hierarchical levels), thereby enhancing the overall level of WSC.

### The relationship between workplace social capital and organizational commitment

This study also identified a significant positive correlation between WSC and OC (*P* < 0.01). From the perspective of social exchange theory, when nurses receive trust from colleagues, collaboration within the team, and support from supervisors, the resulting social capital constitutes a critical relational resource that may be associated with lower emotional exhaustion and greater professional wellbeing and a sense of belonging (Brunetto et al., 2013). Such positive social exchanges, grounded in the principle of reciprocity, may be associated with higher levels of OC among nurses (Chen et al., 2017; Ghazy et al., 2023). Previous studies have demonstrated that high-quality supervisor–employee relationships and effective team collaboration are key predictors of OC (Brunetto et al., 2013; Rodwell et al., 2009). In clinical nursing—an environment that relies heavily on teamwork—interpersonal interactions characterized by trust, cohesion, and knowledge sharing may be associated with better care quality and risk management, and may be associated with stronger nurses' identification with organizational goals (Jafari et al., 2018; Shin and Lee, 2016). Therefore, WSC provides both emotional and instrumental support for nurses and, through social exchange mechanisms, may help explain deeper emotional attachment and OC.

### The relationship between EI and organizational commitment

A significant positive correlation was also observed between EI and OC (*P* < 0.01). EI theory emphasizes that the ability to utilize emotions is a critical determinant of individuals' work attitudes and behaviors (Bulmer Smith, 2009). Nurses with high EI can effectively leverage emotional information to guide constructive actions and make rational decisions when confronted with clinical challenges, thereby enhancing their sense of control and achievement at work (Kahveci et al., 2025; Ranjbar Ezzatabadi et al., 2012). Such positive self-regulation and goal-directed behaviors may be associated not only with greater work engagement but also with stronger nurses' emotional connection to the organization (Zhang et al., 2012). Furthermore, nurses with higher emotional intelligence exhibit greater emotional stability, enabling them to buffer the impact of short-term emotional fluctuations on professional identity and reduce turnover intentions arising from negative emotions (Galanis et al., 2024). Abraham et al. further highlighted that individuals with high EI are more adept at cultivating positive workplace relationships, and these favorable emotions directed toward colleagues and supervisors can extend to broader organizational identification and commitment (Abraham, 2000). Therefore, EI may serve not only as an internal psychological resource that enhances individual performance but also as an emotional resource that may help explain higher OC.

### Workplace social capital as a potential indirect pathway between EI and organizational commitment

Path analysis in the present study revealed that the direct pathway from EI to OC accounted for 38.62% of the total effect (*P* < 0.01), whereas the indirect pathway through WSC accounted for 61.38% (*P* < 0.01), indicating that WSC is an important intervening variable in this relationship. The presence of a significant direct effect suggests that EI, as an inherent emotional capability, has a fundamental association with OC. The stronger indirect pathway, however, suggests that a substantial portion of this association is explained through the development of high-quality interpersonal relationships and the accumulation of social capital. Importantly, because of the cross-sectional design, these findings should be interpreted as statistical associations rather than causal sequences.

Integrating EI theory and social exchange theory provides a deeper understanding of the underlying mechanism. EI theory posits that emotional competencies are central regulatory factors in interpersonal interactions (Schutte et al., 2001). Clinical nurses' EI is theoretically linked to their ability to perceive, express, and manage emotions. These competencies, in turn, have been associated with trust, mutual support, and cohesion within teams, which are core elements of WSC (Xu et al., 2021). Building on this foundation, social exchange theory offers a framework for understanding the transformation of resources and reciprocal behaviors: when nurses leverage high EI to acquire and accumulate social capital—such as supervisor support, coworker trust, and team cohesion—these relational resources have been found to be associated with lower occupational stress, higher job satisfaction and professional efficacy (Berthelsen et al., 2021; Shin and Lee, 2016; Ranjbar Ezzatabadi et al., 2012; Liu et al., 2023), and consistent with the principle of reciprocity, stronger OC. The observed indirect effect is consistent with the hypothesized mediational pathway (EI → WSC → OC), yet the cross-sectional design precludes causal inference regarding this sequence. By foregrounding WSC, this study deepens understanding of the potential theoretical pathway linking WSC to nurses' organizational commitment and may provide theoretical insights for nursing human resource management.

## Conclusion

This study identified significant positive correlations among EI, WSC, and OC. Moreover, WSC exhibited a mediational pattern consistent with partial mediation in this relationship, with the indirect effect surpassing the direct effect. These findings offer empirical grounds for directing managerial attention toward modifiable psychosocial factors in the nursing workforce.

When interpreting these correlational findings for practice, however, it is critical to distinguish between evidence generated by the current study and recommendations informed by existing theory or prior intervention literature. From our data, we can only conclude that EI and WSC are significantly associated with OC and that WSC may represent a potential indirect pathway linking EI to OC, consistent with the hypothesized mediational model. Accordingly, these variables qualify as potential targets for future intervention development. Nursing managers might consider investing in team-building strategies that foster vertical support and horizontal collaboration, as well as programs designed to cultivate emotional competencies—provided that such initiatives are piloted and evaluated rigorously. Nevertheless, because our cross-sectional design does not test causal effects, we caution that implementing EI training or networking initiatives should not be equated with a guaranteed increase in OC. Future longitudinal or quasi-experimental studies are warranted to examine whether deliberate modifications in EI or WSC produce the expected gains in OC before these strategies are adopted at scale.

### Limitations

1. This study employed convenience cluster sampling of clinical nurses from four hospitals in a single province, which may introduce selection bias and limit generalizability. Nurses were nested within hospitals and units, and unaccounted clustering may have resulted in underestimated standard errors, overly narrow confidence intervals, and an increased risk of Type I error. Although leave-one-hospital-out sensitivity analyses were conducted to assess institutional influence, they do not formally account for clustering. Given the small number of hospitals (*n* = 4), multilevel or cluster-robust analyses may yield limited and potentially unstable higher-level inference. Therefore, the reported *P* values and confidence intervals should be interpreted cautiously as exploratory rather than confirmatory evidence. Future studies should include more hospitals across broader geographic regions to enable appropriate statistical adjustment and strengthen inferential robustness.
2. This study utilized a quantitative design, employing self-reported questionnaires to assess participants' abilities, attitudes, and behaviors. Self-reported measures may introduce social desirability bias, which could potentially compromise the accuracy of the findings. Future research could extend these findings by conducting structured or semi-structured qualitative interviews with clinical nurses to explore the variables in greater depth, thereby providing a more comprehensive understanding of participants' characteristics and behaviors.
3. The sample size was based on the conventional 10-cases-per-item heuristic, which does not formally assess power for SEM with mediation. Although the indirect effect was significant based on bootstrap confidence intervals, statistical power and estimate precision remain uncertain. Larger, adequately powered samples are needed to validate these findings.
4. Although the mediating role of WSC was evaluated through theoretical reasoning and statistical testing, it is important to note that the cross-sectional nature of the data precludes definitive causal inference. Future studies could adopt longitudinal or experimental designs, assessing variables at multiple time points to more robustly examine the proposed causal model.
5. Although the associations remained robust after adjustment for measured demographic and occupational characteristics, these covariates were not incorporated into the primary SEM. Thus, residual confounding due to unmeasured or inadequately measured factors cannot be completely excluded. Future studies should incorporate relevant covariates directly into the SEM and use longitudinal designs to further validate these findings.
6. Measurement invariance was examined only across hospital levels, and not across other subgroups such as age and employment; thus, comparability across these groups remains unverified and warrants cautious interpretation. In addition, the SEM was based on an a priori theoretical framework without systematic comparison of competing or alternative models. Therefore, the possibility that other models may also demonstrate good fit cannot be ruled out. Future studies should extend invariance testing across subgroups and compare competing models to further assess the robustness of the findings.

## Data Availability

The raw data supporting the conclusions of this article will be made available by the authors, without undue reservation.
