# Supplementary material for: Association between emotional intelligence and organizational commitment among clinical nurses: workplace social capital as a potential mediator in a cross-sectional study
Source: Front Psychol. 2026 Oct 13;17:1871400. doi: 10.3389/fpsyg.2026.1871400 (PMC13710057; doi:10.3389/fpsyg.2026.1871400)
Supplement: Supplementary file 2 [file Table_2.docx]

**一、Multiple Linear Regression Analysis**

**Appendix 2 Table 1: Multiple Linear Regression Analysis of Organizational Commitment**

| Model | Variable | B | SE | t | P | Collinearity Statistics | |
| --- | --- | --- | --- | --- | --- | --- | --- |
|  |  |  |  |  |  | Tolerance | VIF |
| Model 1 | (Constant) | 20.817 | 2.253 | 9.238 | ＜0.001 | / | / |
|  | EI | 0.203 | 0.029 | 7.088 | ＜0.001 | 0.772 | 1.295 |
|  | WSC | 0.865 | 0.067 | 13.005 | ＜0.001 | 0.772 | 1.295 |
| Model 2 | (Constant) | 15.212 | 4.528 | 3.360 | ＜0.001 | / | / |
|  | EI | 0.167 | 0.029 | 5.818 | ＜0.001 | 0.707 | 1.414 |
|  | WSC | 0.828 | 0.065 | 12.771 | ＜0.001 | 0.741 | 1.349 |
|  | Gender | -1.558 | 1.513 | -1.030 | 0.304 | 0.933 | 1.072 |
|  | Age | 1.699 | 0.575 | 2.953 | 0.003 | 0.573 | 1.744 |
|  | **Hospital Grade** | 1.411 | 1.034 | 1.365 | 0.173 | 0.494 | 2.026 |
|  | Department | -0.131 | 0.156 | -0.843 | 0.400 | 0.978 | 1.023 |
|  | Monthly Income | 0.508 | 0.536 | 0.948 | 0.343 | 0.435 | 2.297 |
|  | Highest Educational Attainment | -0.589 | 0.903 | -0.653 | 0.514 | 0.894 | 1.118 |
|  | Position | 0.848 | 0.619 | 1.369 | 0.171 | 0.715 | 1.398 |
|  | Marital Status | 2.811 | 0.874 | 3.217 | 0.001 | 0.711 | 1.406 |
|  | **Employment Type** | 0.389 | 0.816 | 0.477 | 0.634 | 0.732 | 1.366 |
|  | Night Shift Frequency | -0.304 | 0.309 | -0.983 | 0.326 | 0.744 | 1.344 |
|  | Household Registration | 0.756 | 0.590 | 1.281 | 0.201 | 0.947 | 1.056 |

Note：**Model 1**，R²=0.381，F value=198.889，P<0.001；**Model** 2，R² = 0.446，ΔR²= 0.065，F value =39.267，P<0.001，Durbin-Watson =1.866

**Appendix 2 Figure 1: Residual Diagnostic Plots for the Multiple Linear Regression Model**

二、**Model Fit Results for the Measurement Models of the Three Scales**

**Appendix 2 Figure 2: Measurement Model Diagram of the Workplace Social Capital Scale**

**Appendix 2 Table 2: Fit Indices for the Two‑Factor Measurement Model of the Workplace Social Capital Scale**

| **Index** | **Reference Value** | **Measured Value** | **Conclusion** |
| --- | --- | --- | --- |
| χ^2^/df | ＜5 | 5.516 | marginal fit |
| RMSEA | ＜0.08 | 0.08 | Acceptable Fit |
| SRMR | ＜0.08 | 0.024 | Good fit |
| NFI | ＞0.9 | 0.982 | Good fit |
| IFI | ＞0.9 | 0.986 | Good fit |
| TLI | ＞0.9 | 0.973 | Good fit |
| CFI | ＞0.9 | 0.986 | Good fit |
| GFI | ＞0.9 | 0.971 | Good fit |

**Appendix 2 Table 3: Construct Validity of the Workplace Social Capital Scale**

| **Construct** | **Cr** | **AVE** | **sqrt(AVE)** | **link** | **nonlink** |
| --- | --- | --- | --- | --- | --- |
| link | 0.954 | 0.874 | 0.935 | 0.935 | / |
| nonlink | 0.888 | 0.626 | 0.791 | 0.711** | 0.791 |

Note：**, P＜0.001

**Appendix 2 Figure 3: Measurement Model Diagram of the Organizational Commitment Scale**

**Appendix 2 Table 4: Fit Indices for the Three‑Factor Measurement Model of the Organizational Commitment Scale**

| **Index** | **Reference Value** | **Measured Value** | **Conclusion** |
| --- | --- | --- | --- |
| χ^2^/df | ＜5 | 3.804 | Good fit |
| RMSEA | ＜0.08 | 0.066 | Good fit |
| SRMR | ＜0.08 | 0.062 | Good fit |
| NFI | ＞0.9 | 0.920 | Good fit |
| IFI | ＞0.9 | 0.940 | Good fit |
| TLI | ＞0.9 | 0.926 | Good fit |
| CFI | ＞0.9 | 0.940 | Good fit |
| GFI | ＞0.9 | 0.916 | Good fit |

**Appendix 2 Table 5: Construct Validity of the Organizational Commitment Scale**

| **Construct** | **Cr** | **AVE** | **sqrt(AVE)** | **NC** | **CC** | **AC** |
| --- | --- | --- | --- | --- | --- | --- |
| NC | 0.861 | 0.51 | 0.714 | 0.714 | / | / |
| CC | 0.753 | 0.358 | 0.598 | 0.424** | 0.598 | / |
| AC | 0.888 | 0.581 | 0.762 | 0.726** | 0.32** | 0.762 |

Note：**, P＜0.001

**Appendix 2 Figure 4: Measurement Model Diagram of the Emotional Intelligence Scale**

**Appendix 2 Table 6: Fit Indices for the Four‑Factor Measurement Model of the Emotional Intelligence Scale**

| **Index** | **Reference Value** | **Measured Value** | **Conclusion** |
| --- | --- | --- | --- |
| χ^2^/df | ＜5 | 4.62 | Good fit |
| RMSEA | ＜0.08 | 0.075 | Good fit |
| SRMR | ＜0.08 | 0.045 | Good fit |
| NFI | ＞0.9 | 0.927 | Good fit |
| IFI | ＞0.9 | 0.942 | Good fit |
| TLI | ＞0.9 | 0.929 | Good fit |
| CFI | ＞0.9 | 0.942 | Good fit |
| GFI | ＞0.9 | 0.919 | Good fit |

**Appendix 2 Table 7: Construct Validity of the Emotional Intelligence Scale**

| **Construct** | **Cr** | **AVE** | **sqrt(AVE)** | **OEA** | **UOE** | **ROE** | **SEA** |
| --- | --- | --- | --- | --- | --- | --- | --- |
| OEA | 0.882 | 0.653 | 0.808 | 0.808 | / | / | / |
| UOE | 0.87 | 0.626 | 0.791 | 0.592** | 0.791 | / | / |
| ROE | 0.885 | 0.659 | 0.812 | 0.458** | 0.738** | 0.812 | / |
| SEA | 0.84 | 0.572 | 0.756 | 0.339** | 0.34** | 0.328** | 0.756 |

Note：**, P＜0.001

**三、Measurement invariance testing**

**Appendix 2 Table 8: Measurement invariance testing results**

| Model | χ² | df | CFI | RMSEA (90% CI) | SRMR | Model Comparison 1 vs 2 | | | Decision |
| --- | --- | --- | --- | --- | --- | --- | --- | --- | --- |
|  |  |  |  |  |  | Δχ² (Δdf) | ΔCFI | ΔRMSEA |  |
| Configural | 141.392 | 48 | 0.955 | 0.055 [0.045, 0.065] | 0.042 | / | / | / | / |
| Metric | 149.559 | 54 | 0.954 | 0.052 [0.042, 0.062] | 0.047 | 8.168 (6) | 0.001 | -0.003 | Accept |

**四、Leave‑One‑Out Sensitivity Analysis**

**Leave‑One‑Out Sensitivity Analysis: Regression Coefficients and Significance of Core Predictors After Excluding Each Hospital**

**Excluding 70 Respondents from Hospital A**

**Excluding 81 Respondents from Hospital B**

**Excluding 81 Respondents from Hospital B**

**Excluding 197 Respondents from Hospital D**

**648 Respondents from the Four Hospitals (A, B, C, and D)**

**Appendix 2 Table 9: Summary of Leave‑One‑Out Sensitivity Analysis Results**

| Model |  | Beta | t | P | Partial Correlation | Tolerance |
| --- | --- | --- | --- | --- | --- | --- |
| ABCD | EI | 0.403^b^ | 11.407 | <0.001 | 0.412 | 0.879 |
|  | WSC | 0.527^b^ | 16.679 | <0.001 | 0.552 | 0.921 |
| Excluding A | EI | 0.388^b^ | 10.228 | <0.001 | 0.395 | 0.891 |
|  | WSC | 0.486^b^ | 14.304 | <0.001 | 0.516 | 0.966 |
| Excluding B | EI | 0.449^b^ | 12.253 | <0.001 | 0.462 | 0.890 |
|  | WSC | 0.582^b^ | 17.030 | <0.001 | 0.586 | 0.854 |
| Excluding C | EI | 0.357^b^ | 7.296 | <0.001 | 0.370 | 0.899 |
|  | WSC | 0.512^b^ | 11.469 | <0.001 | 0.531 | 0.900 |
| Excluding D | EI | 0.403^b^ | 9.455 | <0.001 | 0.412 | 0.831 |
|  | WSC | 0.549^b^ | 15.181 | <0.001 | 0.587 | 0.914 |

**Appendix 2 Table 10：Modification Indices**

|  |  | M.I. | Par Change |
| --- | --- | --- | --- |
| e8<-->e9 |  | 9.815 | -1.356 |
| e7<-->e10 |  | 6.96 | -1.127 |
| e7<-->e8 |  | 10.593 | 1.807 |
| e5<-->e8 |  | 4.48 | 0.731 |
| e4<-->e10 |  | 12.992 | 1.444 |
| e4<-->e7 |  | 9.594 | -1.49 |
| e2<-->e9 |  | 5.677 | -0.771 |
| e2<-->e7 |  | 9.315 | 1.263 |
| e2<-->e4 |  | 5.354 | -0.889 |
| e1<-->e10 |  | 6.27 | -1.04 |
| e1<-->e4 |  | 6.258 | 1.172 |

**Appendix 2 Table 11：Standardized Residual Covariances**

|  | AOC | COC | NOC | LINKING | NONLINKING | SEA | ROE | UOE | OEA |
| --- | --- | --- | --- | --- | --- | --- | --- | --- | --- |
| AOC | 0 |  |  |  |  |  |  |  |  |
| COC | -0.733 | 0 |  |  |  |  |  |  |  |
| NOC | 0.045 | 1.985 | 0 |  |  |  |  |  |  |
| LINKING | 0.295 | -0.638 | -0.923 | 0 |  |  |  |  |  |
| NONLINKING | 0.177 | 0.999 | -0.971 | 0 | 0 |  |  |  |  |
| SEA | 1.713 | 1.33 | -0.466 | 2.495 | 2.45 | 0 |  |  |  |
| ROE | 0.037 | 0.521 | 0.458 | 0.329 | 0.533 | -0.497 | 0 |  |  |
| UOE | -0.595 | 0.325 | 1.152 | -0.145 | -0.244 | -0.803 | 0.128 | 0 |  |
| OEA | -0.398 | 1.208 | 0.484 | -1.963 | -1.428 | 1.697 | -0.579 | 0.375 | 0 |
