# Supplementary material for: Association between emotional intelligence and organizational commitment among clinical nurses: workplace social capital as a potential mediator in a cross-sectional study
Source: Front Psychol. 2026 Oct 13;17:1871400. doi: 10.3389/fpsyg.2026.1871400 (PMC13710057; doi:10.3389/fpsyg.2026.1871400)
Supplement: Supplementary file 3 [file Table_1.docx]

**Appendix 1:** Results of the unmeasured latent method factor analysis for common method bias.
